# Pre-hospital Care and Delay in Patients with Traumatic Brain Injury in a Tertiary Care Center: A Descriptive Cross-sectional Study

**DOI:** 10.31729/jnma.8629

**Published:** 2024-07-31

**Authors:** Milan Regmi, Om Prakash Bhatta, Mohan Raj Sharma

**Affiliations:** 1 Panchkhal Primary Health Care Center, Panchkhal, Kavrepalanchowk, Nepal; 2 Nisarga Hospital and Research Center, Dhangadhi, Kailali, Nepal; 3 Department of Neurosurgery, Tribhuvan University Teaching Hospital, Institute of Medicine, Maharajgunj, Kathmandu, Nepal

**Keywords:** *brain injuries*, *continuity of patient care*, *time-to-treatment*

## Abstract

**Introduction:**

Timely institution of pre-hospital therapies aimed at damage control and the appropriately timed decision of transfer to higher centers for definitive neurosurgical management are crucial in determining the outcome of patients following traumatic brain injury. This study aimed to evaluate the factors determining pre-hospital care and delay in patients with traumatic brain injury.

**Methods:**

This was a descriptive cross-sectional study conducted in a tertiary care center after obtaing ethical approval from the Institutional Review Board (approval number 392 (6-11) E2). All patients with traumatic brain injury who presented to the emergency department from 1 July, 2018 to 15 June, 2019 were enrolled. Data related to patient demographics, the primary cause of the incident, grading of traumatic brain injury on admission, pre-hospital care, and variables that cause pre-hospital delay were collected.

**Results:**

In this study of 144 patients with traumatic brain injury, we found that 70 (48.61%) experienced transfer delays exceeding one hour. There were 71 (49.31%) patients aged 15-44 years, and 100 (69.44%) were male, with falls being the primary cause of 119 (82.64%). Most patients had mild traumatic brain injury 80 (55.56%). Out of 144, 20 (13.89%) received prehospital care, and 28 (19.44%) underwent a computed tomography scan of the head before arrival.

**Conclusions:**

Our study highlights the challenges in pre-hospital care and delays in reaching for neurosurgical care in patients with traumatic brain injury. Falls, road accidents, and physical assaults were the leading causes.

## INTRODUCTION

Traumatic Brain Injury (TBI), often called the "silent epidemic," is a major public health concern, posing significant challenges to society as the leading cause of death and disability worldwide among all traumatic injuries.^1,2^ Although studies have demonstrated that the lifetime incidence of TBI in developed nations is 500-800 new cases per 100,000 people annually, such studies are scarce in low- and middle-income countries (LMICs). These countries, affected mainly by the morbidity and mortality associated with TBI, face a double burden of increased injuries and poor outcomes due to resource constraints.^3^

The third most common cause of mortality in Nepal is TBI, accounting for 8% of all deaths.^4^ Although mortality has decreased significantly, disability due to TBI remains a significant challenge. Therefore, improving pre-hospital care, minimizing pre-hospital delays, and developing advanced neurosurgical facilities to reduce in-hospital delays are crucial for improving patient outcomes. It is also crucial to know the current scenario of pre-hospital delay in our scenario.

This study aimed to evaluate pre-hospital care, and pre-hospital delays in receiving definitive neurosurgical treatment for patients with TBI.

## METHODS

This descriptive cross-sectional study included all TBI patients referred to the Neurosurgery Department from the Emergency Department of Tribhuvan University Teaching Hospital (TUTH), Kathmandu, between 1 July 2018 to 15 June 2019, who fulfilled the inclusion criteria. Approval from the Institutional Review Board of Institute of Medicine(Reference number: 392 (611) E2) was obtained before data collection. Data were collected from the patient's visitors or relatives, ambulance driver, person accompanying the patient to the emergency department (ED), emergency admission ticket, and doctors on duty. A self-structured proforma was filled out with all the necessary details, and follow-up of the cases was conducted until the neurosurgery department made the final decision. Informed consent was obtained from all the patients or their caregivers before enrollment in the study. This study was conducted following the principles outlined in the Declaration of Helsinki. ^5^

All patients who sustained a traumatic brain injury were admitted to a Tribhuvan University Teaching Hospital (TUTH) emergency department and excluded all patients or their relatives who denied giving informed written consent.

Data related to patient demographics (age and sex), primary causes of the incident, prehospital care received (such as neck stabilization, supplemental oxygen administration or fluid administration before reaching any health center from the site of injury), type of initial health care facility visited, timing of referral from the primary center to the tertiary care hospital, mode of transfer, grading of TBI upon admission based on GCS, time to perform a Computed Tomogrpahy scan, and the timing of neurosurgical consultation upon arrival at the hospital were collected. The TBI was graded in the ED based on the Glasgow Coma Scale (GCS) and categorized as mild (GCS 13-15), moderate (GCS 9-12), or severe (GCS 3-8).

The study variables were obtained and recorded using a proforma. Data were entered into the Statistical Package for the Social Sciences (SPSS) version 25, and descriptive statistical analysis was conducted to determine the frequency and percentage of binary data.

## RESULTS

A total of 144 patients with TBI, ranging in age from 2 - 84 years were enrolled in the study among which 71(49.31%) of the patients were aged 15 - 44 years, and 100 (69.44%) were male (Table 1).

**Table 1:** Distribution of patients with TBI by age groups (n=144).

Age groups (years)	n (%)
0-14	46 (31.94)
15-29	40 (27.78)
30-44	31 (21.53)
45-59	16 (11.11)
>60	11 (7.64)
**Sex**	
Male	100 (64.44)
Female	44 (33.56)

Regarding the severity of the injury, mild TBI was 80 (55.56%), moderate was 35 (24.31%) and severe was 29 (20.14%), as determined by the Glasgow Coma Scale. The primary cause of TBI were falls, accounting for 119 (82.64%) cases, motor vehicle accidents 21 (14.58%), physical assault in 4 (2.78%). Alcohol intoxication during the incident was found in 28 (19.44%) patients. In terms of prehospital care and primary care referral, 20 (13.89%) of the patients received prehospital care, which is any form of treatment, such as neck stabilization, supplemental oxygen administration or fluid administration, provided by paramedics, ambulance drivers, or health workers, before reaching any health center from the site of injury. Among 144, 85 (59.03%) patients reached the tertiary center from the primary center in less than one day. CT scans were done before reaching the tertiary center in 28 (19.44%) of cases (Table 2).

**Table 2:** Time taken by a patient with TBI to reach the tertiary center from the site of injury (n=144).

**Time taken**	**n (%)**
Time taken by a patient with TBI to reach the tertiary center from the site of injury	
<1 day	85 (59.03)
>1 day to <7 days	39 (27.08)
>7 days	20 (13.89)
Time taken by a patient with TBI to reach any health care setting center from the site of injury	
0-15 minutes	45 (31.25)
16-45 minutes	20 (13.89)
About 1 hour	9 (6.25)
More than 1 hour	70 (48.61)
Time taken to do CT scan in a patient with TBI at the tertiary center	
30 minutes	78 (54.17)
30-60 minutes	37 (25.69)
>60 minutes	29 (20.14)

## DISCUSSION

TBI is a significant cause of preventable trauma-related mortality in Nepal and affects the young population, leading to substantial socioeconomic consequences. As TBI is a time-sensitive illness, the timing of hospital presentation and initiation of definitive neurosurgical therapy are critical for prognosis and mortality. Prehospital care, including resuscitative measures at the injury scene and during transport, is essential to prevent secondary brain injuries and other high-risk complications, such as hypoxia, hypotension, and expanding intracranial mass lesions.^4 6^

Falls were identified as the primary cause of TBI, accounting for the most cases, in contrast to studies from other countries,where road traffic accidents (RTAs) were the primary cause. This difference may be related to lifestyle and occupation, as a significant proportion of Nepal's population and the area surrounding the study site live in rural areas with mountains and hills, where activities such as climbing trees are important in agriculture. Additionally, inadequate infrastructure with poor safety standards, particularly in the construction and transportation sectors, contribute to common falls from heights,which are a major occupational hazard. This underscores the need for effective prevention strategies to target hazards and minimize risk-taking activities, particularly among children and older people.^3,7^ As RTAs also lead to a high proportion of TBI cases, targeted prevention with a focus on car safety, traffic education, road infrastructure improvements, and traffic regulation enforcement is key. Promoting the use of seatbelts, helmets, and child restraints, along with strict laws against impaired and distracted driving, can significantly decrease the incidence of TBI.^6^ The most frequent causes of TBI in the United States are high-speed vehicle accidents,violence, sports-related incidents,and injuries at construction sites.^8,9^ Whereas in a study conducted in Nepal, the top three causes of TBI were falling from heights, road traffic accidents (RTAs), and violence.^4,10^

However, a study conducted in a multispecialty private hospital involving 167 patients with TBI found RTAs as the most common mechanism of injury (59%), followed by falls (32%), and physical assault (9%).^10^ In a study conducted in Uganda involving 3749 patients, road traffic injuries (88.9%) and falls (11.1%) were the major causes of TBI.^11^ Another study conducted in Pakistan showed similar findings, with TBI predominantly caused by road traffic accidents (62.6%), followed by falls (31.7%), and physical assault (5.5%).^12^

In a similar study, 75.7% of patients were transferred from other hospitals, of whom 30 (56.6%) had visited one hospital, and 17 (32%) had visited two hospitals before reaching tertiary care centers with neurosurgical facilities. The unavail ability of neurosurgery facilitieswas the main reason for transfer out (77.4%). The mortality and length of hospital stay trends were higher in the groupwho hadvisited other centers before coming to the tertiary center compared to those who came directly. ^13^

The demographic distribution of TBI patients revealed a higher representation of younger age groups, particularly individuals aged 0-14 years and 15-29 years, highlighting their vulnerability to TBI incidents. Other studies conducted in Uganda showed that 70% of patients were between 19 and 45 years of age, while in Pakistan, the most common age group was 21-30 years, with 470 (34.1%) patients, followed by 331 (24.02%) patients between 31-40 years.^11,12^ This susceptibility can be attributed to factors such as participation in high-risk activities and increased exposure to injury-causing situations. The higher prevalence of TBI among male aligns with previous research, suggesting higher risk-taking behavior or differences in occupational hazards. ^4,14^

Severity assessment using the Glasgow Coma Scale showed that most TBI cases were classified as mild, followed by moderate and severe, consistent with the global distribution of TBI severity. However, it is essential to consider the limitations of the Glasgow Coma Scale as the sole indicator of injury severity.^15^

Major extracranial injuries in a significant proportion of patients indicate the complexity and potential impact of multi-system trauma in TBI cases. Managing these additional injuries should be considered in the overall treatment approach for patients with TBI to ensure comprehensive care and optimize the outcomes.^16,17^

Most patients with TBI are transported to hospitals via ambulance, and timely hospital arrival remains challenging in low and middle-income countries (LMICs). Previous studies have identified various factors that contribute to delays, including selftreatment practices, primary care visits, and inadequate referral systems.^18-21^ These findings emphasize the necessity for well-structured trauma systems that prioritize swift transportation to trauma hospitals. Future interventions should focus on educating patients about the time-sensitive nature of TBI.

Improving pre-hospital care and reducing delays in reaching tertiary care centers are crucial for better patient outcomes. Strengthening pre-hospital emergency medical services and increasing public awareness regarding early medical interventions are essential.^17^ The variations in transfer times from the injury site to the hospital indicate the need for efficient ambulance services and improved transportation infrastructure.

Timely performance of diagnostic procedures, such as CT scans, is critical for accurate diagnosis and treatment planning. Efforts should be directed toward reducing the time taken to perform CT scans after arrival at the tertiary center and time to neurosurgical consultation for timely initiation of definitive management.^17^ Prior studies have shown a 50% decrease in mortality in patients who underwent craniotomy or hematoma drainage within four hours of arrival to the emergency department, highlighting the importance of timely clinical assessment and neurosurgical intervention in patients with TBI.

This study has some limitations that should be considered. First, its reliance on a single tertiary care center limits the generalizability of the findings to broader populations and other healthcare settings. The study's cross-sectional nature restricts causal inference between variables, offering associations rather than definitivecause-effect relationships. Importantvariables, such as socioeconomic status, detailed pre-hospital care specifics, and operative variables, were not uniformly available, potentially affecting the depth of analysis. Moreover, the possibility of recall bias among patients or caregivers regarding events leadingtoTBI or pre-hospital care details cannot be overlooked. A major challenge was obtaining precise injury-to-arrival data for patients with severeTBI who lack accurate responses or available family members. These limitations suggest directions for future research to address these gaps comprehensively.

## CONCLUSIONS

This study identifies falls from heights, road traffic accidents, and physical assault as primary causes of TBI in Nepal. Factors contributing to delayed presentation to neurosurgical care include major external injuries, alcohol intoxication, incidents in public places, and lack of pre-hospital care. Significant delays were observed in reaching the primary care center and referral to tertiary care centers.
